# Revealing the Non-Arrhenius Migration of Oxygen Vacancies at the CeO_2_(111) Surface

**DOI:** 10.1021/acs.jpclett.5c00444

**Published:** 2025-04-06

**Authors:** Yujing Zhang, Huabing Cai, Beien Zhu, Zhong-Kang Han, Hui Li, M. Verónica Ganduglia-Pirovano, Yi Gao

**Affiliations:** † Beijing Advanced Innovation Center for Soft Matter Science and Engineering, Beijing University of Chemical Technology, Beijing 100029, China; ‡ Photon Science Research Center for Carbon Dioxide, Shanghai Advanced Research Institute, Chinese Academy of Sciences, Shanghai 201210, China; § School of Materials Science and Engineering, Zhejiang University, Hangzhou 310027, China; ∥ Instituto de Catálisis y Petroleoquímica, Consejo Superior de Investigaciones Científicas, 28049 Madrid, Spain; ⊥ Key Laboratory of Low-Carbon Conversion Science & Engineering, Shanghai Advanced Research Institute, Chinese Academy of Sciences, Shanghai 201210, China

## Abstract

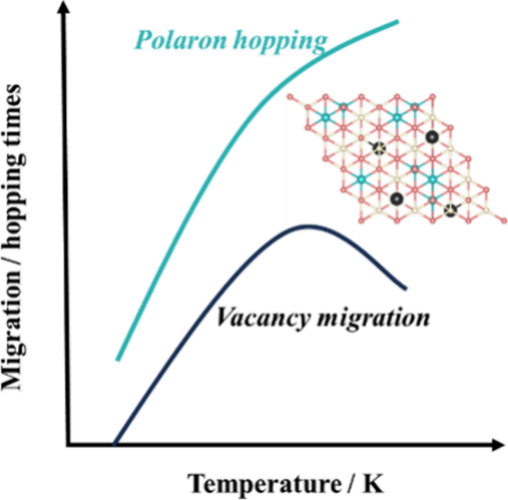

Ceria (CeO_2_) is renowned for its exceptional oxygen storage capacity, which makes it highly valuable in various applications. Central to its functionality is the migration of oxygen vacancies (V_O_’s). While previous studies have extensively examined the distribution of V_O_’s and Ce^3+^ polarons, their kinetic behaviors and interactions, especially in the presence of multiple vacancies, are not yet fully understood. In this study, we employ density functional theory (DFT), ab initio molecular dynamics (AIMD) simulations, and machine-learning methods to investigate these phenomena. Our findings reveal a nonmonotonic temperature dependence of the migration rate of V_O_’s at the CeO_2_(111) surface. Our theoretical model further demonstrates that the migration of V_O_’s and the hopping of polarons are intricately coupled. Notably, frequent polaron hopping at high temperatures hinders V_O_ migration, indicating a non-Arrhenius mechanism. This finding is further validated through long-time molecular dynamics simulation enhanced with neural network potentials. Our results provide a microscopic understanding of the interplay between V_O_’s and Ce^3+^ polarons, offering crucial insights into the complex dynamics governing oxygen vacancy migration in ceria. This knowledge paves the way for improved material design and functionality.

Ceria exhibits extraordinary properties in oxygen storage, release, and redox catalytic performance, making it widely used in applications such as fuel cells,^1,2^ resistance switching devices,^3^ automobile exhaust gas treatment,^4−7^ hydrogen production,^8^ and biology.^9,10^ A key factor in these applications is the role of oxygen vacancies (V_O_’s), which generate excess electrons that reduce Ce^4+^ to Ce^3+^. This phenomenon has sparked significant interest in the distribution of V_O_’s and excess charges at the CeO_2_(111) surface.^11−22^

Extensive research combining experimental characterization and theoretical calculations has advanced our understanding of these aspects. Studies focusing on low oxygen vacancy (V_O_) concentrations and/or focusing on the surface and subsurface layers have established that V_O_’s at the CeO_2_(111) surface are mainly distributed in the subsurface, with excess electrons forming Ce^3+^ polarons localized on next-nearest neighbor (NNN) cationic sites.^11,13^ However, a recent study suggested that V_O_’s may occupy the third oxygen layer at higher vacancy concentrations.^22^ Under reducing conditions, polarons readily associate with V_O_’s to form polaron–vacancy complexes, affecting the activation energy of electron hopping and V_O_ migration.^23−27^

Polaron electron transport in bulk ceria has been experimentally observed, with Tuller and colleagues reporting activation energies of 0.40–0.52 eV.^28^ However, the migration of V_O_’s at the CeO_2_(111) surface remains contentious.^11,29,30^ Previous theoretical studies have primarily focused on specific electron configurations to analyze migration pathways.^23,31−33^ Li et al. initially proposed a two-step exchange mechanism for V_O_ migration at the CeO_2_(111) surface, involving surface-to-subsurface and subsurface-to-surface exchange, with a notably lower energy barrier compared to direct surface hopping processes.^31^ Subsequent studies have highlighted the influence of polaron positions on V_O_ migration.^23,32,33^ Su et al. introduced a “polaron hopping-assisted mechanism” for surface V_O_ migration involving two nearest neighbor (NN) Ce^3+^ ions,^33^ while Zhang et al. proposed two mechanisms: the “NN polaron-hindered mechanism” and the “NN polaron-promoted mechanism”, wherein temperature significantly impacts V_O_ and polaron behaviors.^34^ Despite these insights into single-V_O_ migration at the CeO_2_ surface, the kinetic behavior and interaction between V_O_’s and polarons, particularly in the presence of multiple V_O_’s, remain poorly understood. Given the natural abundance of V_O_’s in CeO_2_, their interplay with polarons complicates vacancy migration dynamics.

In this study, using four V_O_’s at the CeO_2_(111) surface with 4 × 4 periodicity as a model system, we integrate density functional theory (DFT) calculations, ab initio molecular dynamics (AIMD), and neural network potential (NNP)-enhanced molecular dynamics simulations to elucidate the kinetics of V_O_’s and polarons at varying temperatures. Our findings reveal a strong coupling between V_O_ migration and polaron hopping, resulting in a non-Arrhenius mechanism governing V_O_ migration. Notably, V_O_ migration does not simply increase with temperature, as frequent polaron hopping disrupts preferred migration pathways, consistent with recent experimental observations.^35^ The high polaron concentration stabilizes V_O_’s, with their jumps effectively limiting V_O_ mobility.

All trajectories originate from an identical initial configuration, where four V_O_’s (V_Oa_, V_Ob_, V_Oc_, and V_Od_) are positioned within the subsurface, arranged in a 2 × 2 array. Simultaneously, all Ce^3+^ ions are located in the outermost cerium layer, as illustrated in Figure 1. While this Ce^3+^ arrangement corresponds to a low-energy state, it is not the most stable configuration.^22^ At 300 K, no V_O_ migration occurred within 50 ps, whereas Ce^3+^ polarons underwent 55 hopping events. Polarons in nearest neighbor (NN) positions relative to V_O_’s frequently transitioned to the second cerium layer, occupying a next-nearest neighbor (NNN) position (Figure S1). This observation aligns with prior research indicating that Ce^3+^ ions tend not to be adjacent to V_O_’s.^16^

**Figure 1:**
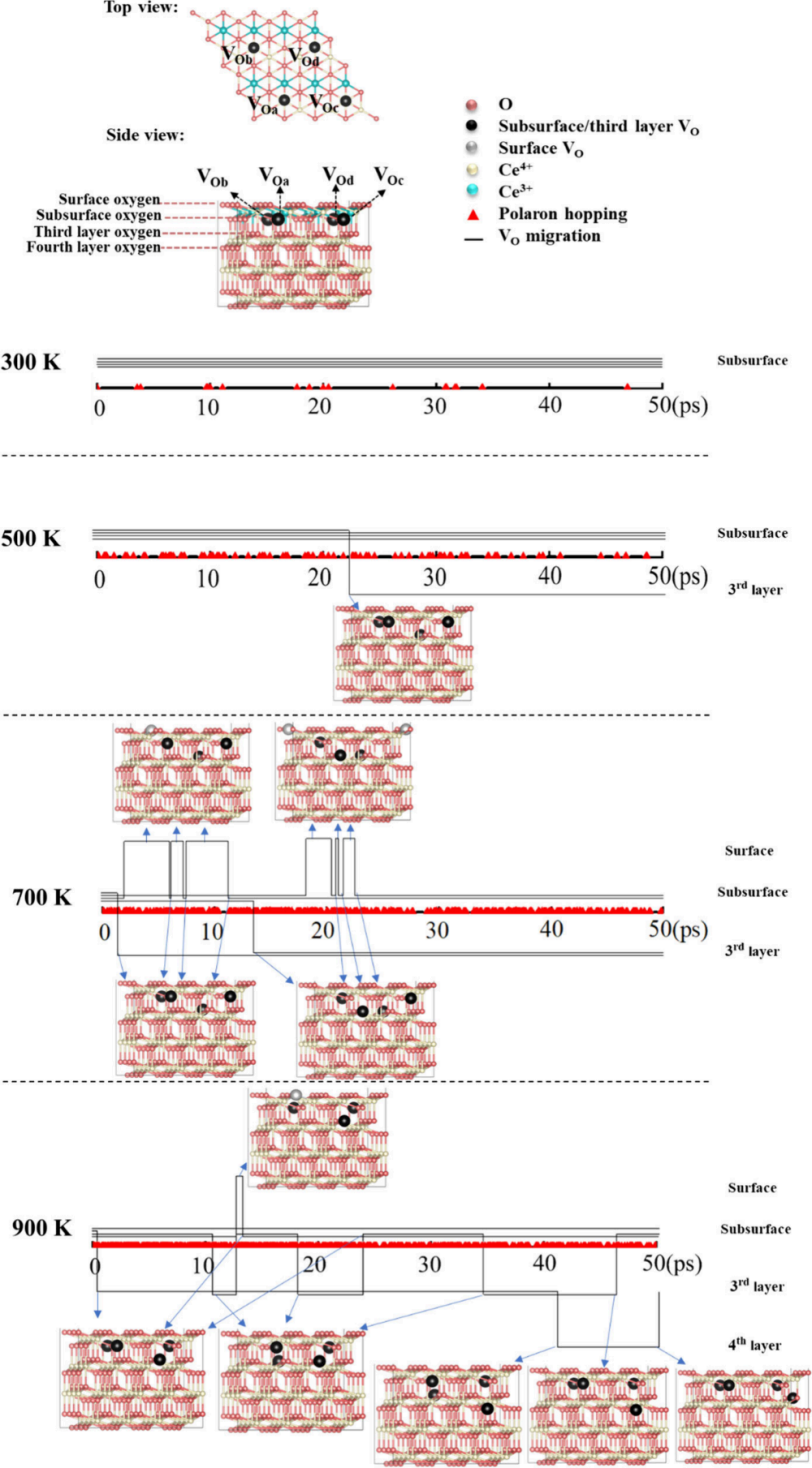
Temperature-dependent trajectories of V_O_ migration and polaron hopping events on the CeO_2_(111) surface. The figure illustrates the movement patterns of V_O_’s and Ce^3+^ polarons at 300, 500, 700, and 900 K, highlighting the coupling between V_O_ migration and polaron hopping.

At 500 K, polarons hopped 347 times and V_Od_ migrated once from the subsurface to the third oxygen layer (Figure 1). At 700 K, migration increased significantly, with 14 migration events observed alongside 1442 polaron hopping events. Initially, V_Od_ followed a migration pattern similar to that at 500 K, moving from the subsurface to the third layer. Subsequently, V_Ob_ migrated six times between the subsurface and the surface, followed by V_Oa_ migrating from the subsurface to the third layer. Finally, V_Oc_ migrated six times between the subsurface and the surface. By the end of the simulation, V_Oa_ and V_Od_ remained in the third layer while V_Ob_ and V_Oc_ settled back in the subsurface.

As the temperature increased to 900 K, the number of polaron hopping events increased sharply to 2393, while V_O_ migration remained relatively limited at 11 occurrences. Notably, V_Oc_ migrated from the subsurface to the third layer, where it remained for an extended period (∼40 ps). It then moved to the fourth layer, staying there for approximately 8 ps before returning to the third layer. Additionally, V_Ob_ migrated six times between the subsurface and the third layer within 50 ps, while V_Oa_ exhibited only two migration events between the subsurface and the surface. It is important to note that the 50 ps AIMD simulations in this study were exploratory rather than aimed at achieving thermodynamic equilibrium. Our primary objective was to determine whether multivacancy configurations exhibit the same migration mechanisms as single-vacancy systems on the CeO_2_(111) surface over picosecond time scales, prioritizing mechanistic insights over strict convergence validation. AIMD simulations reveal that specific migration pathways in multivacancy configurations retain mechanistic features for single-vacancy migration.^34^ In Figure 2A, when a Ce^3+^ occupies a NN position relative to a subsurface V_O_ (V_Oa_), as indicated by the green dotted circle, the relative instability of this configuration drives V_Oa_ to migrate to the surface. During this migration, V_Oa_ crosses a Ce^4+^–Ce^4+^ bridge, resulting in the Ce^3+^ becoming a next-nearest neighbor (NNN). This rearrangement stabilizes the configuration by 0.23 eV. Notably, the activation energy for this migration is remarkably low (0.03 eV), aligning with the proposed NN polaron-promoted mechanism for a single V_O_.^34^ Conversely, the migration of a V_O_ from the surface to the subsurface follows the NN polaron-hindered mechanism.^34^ In this case, the barrier for oxygen atoms for crossing a Ce^4+^–Ce^4+^ bridge (0.25 eV, Figure 2B) is lower than that for crossing a Ce^3+^–Ce^4+^ bridge (0.53 eV, Figure S2). Additionally, after migration through the Ce^4+^–Ce^4+^ bridge (Figure 2B), the positional relationship between V_Oa_ and the Ce^3+^ evolves from NN to NNN (indicated by the green dotted circle).

**Figure 2:**
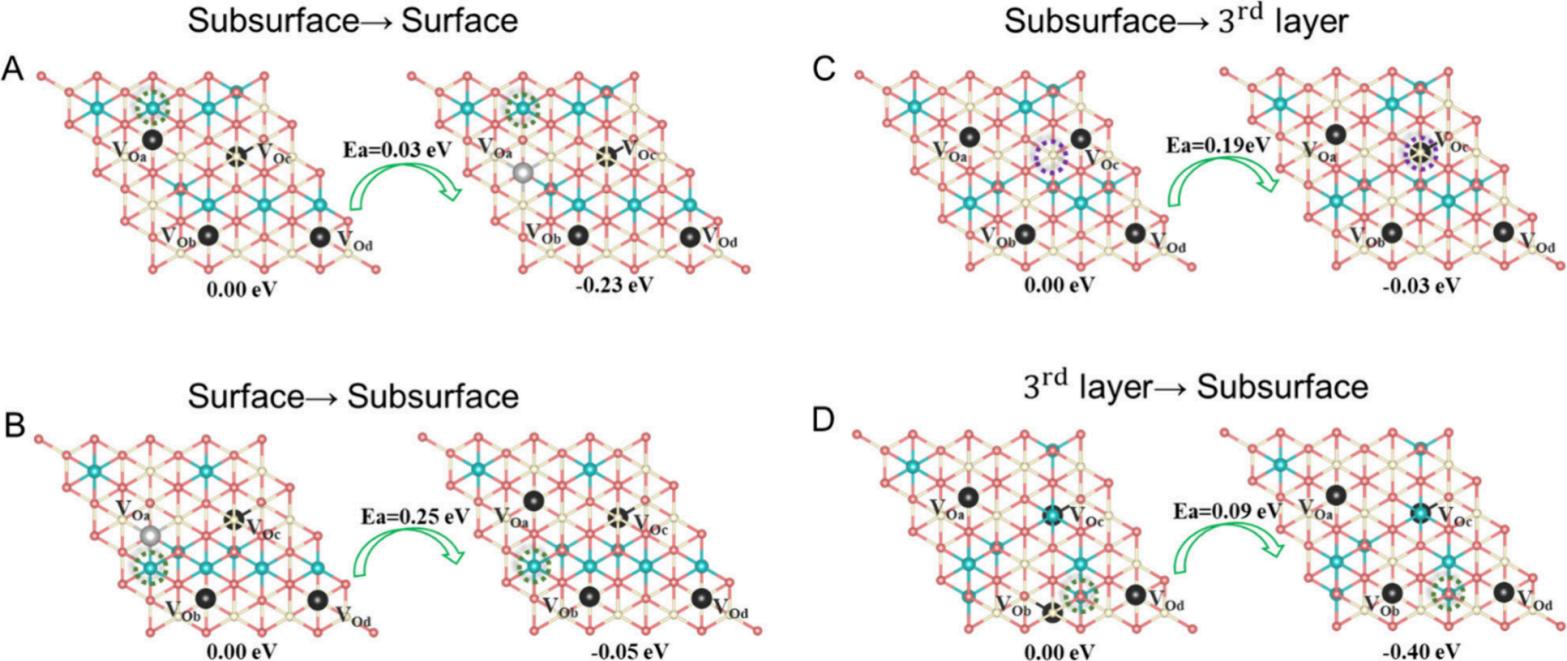
Mechanisms of V_O_ migration. Ce^4+^, Ce^3+^, oxygen atoms, and the surface oxygen vacancy are colored white, blue, pink, and light gray, respectively, while subsurface and third oxygen layer vacancies are colored black, with green dotted circles indicating NN and NNN sites of Ce^3+^ in the initial and final state, respectively. Purple dotted circles indicate Ce^4+^.

Furthermore, we observed that V_O_ migration extends between the subsurface and the third oxygen atomic layer, a phenomenon not reported in single-V_O_ cases.^34^ As illustrated in Figure 2C, the initial configuration consists of a 2 × 2 array of subsurface V_O_’s, where each V_O_ is associated with one surface NNN Ce^3+^ ion and one subsurface (second cationic plane) NNN Ce^3+^ ion, as shown in Figure 2C and Figure S3. When the subsurface V_Oc_ overcomes an energy barrier of 0.19 eV, it migrates to the third layer, where it is coordinated with three Ce^3+^ cations in NNN positions within the subsurface. According to our recent study,^22^ this distinct configuration enhances the stability of third-layer V_O_’s compared to subsurface V_O_’s. This stabilization arises from the additional lattice relaxation of the surface Ce^4+^ ion (highlighted by the purple dotted circle in Figure 2C) located above the third-layer V_O_. As shown in Figure S3, the displacement of this Ce^4+^ ion increases by 0.32 Å between the initial and final configurations in Figure 2C. Moreover, similar to how a subsurface V_O_ is promoted to the surface in the presence of a NN Ce^3+^ ion, V_Ob_ in the third oxygen layer, when located near a Ce^3+^ (indicated by the green dotted circle in Figure 2D), can migrate back to the subsurface layer, leading to a stabilization of 0.40 eV. Additionally, other V_O_ migration pathways from the third layer to the subsurface may arise due to the complexity of the Ce^3+^ configurations in systems with multiple V_O_’s, as illustrated in Figure S4.

In addition, as the temperature increased from 500 to 900 K, subsurface V_O_’s migrate to the third layer, forming a distinct pattern with surrounding subsurface V_O_’s. The distance between the subsurface V_O_ and third-layer V_O_ is 6.11Å, corresponding to the fifth V_O_–V_O_ neighbor distance in bulk CeO_2_, as shown in Figure S5. This configuration was previously identified as a key feature in stabilizing near-surface V_O_ structures on the CeO_2_(111) surface (V_O2_V_O3__5N in ref (22)) in our earlier study.^22^ These configurations remain stable for extended periods: ∼27 ps at 500 K, ∼37–50 ps at 700 K, and ∼34 ps at 900 K.

In our previous study on single-V_O_ migration,^34^ migrations between the surface and subsurface layers followed the NN polaron-hindered mechanism, in which V_O_’s moved across Ce^4+^–Ce^4+^ pairs. Additionally, migrations from the subsurface to the surface predominantly followed the NN polaron-promoted mechanism. In this case, migrations originated from a configuration with a nearest neighbor polaron, with the V_O_ consistently moving away from it across a Ce^4+^–Ce^4+^ pair. The greater stability of the subsurface V_O_, where excess electrons localize on NNN surface cerium positions relative to the vacancy, compared to an equivalent surface V_O_ configuration, is attributed to a larger relaxation energy gain associated with the subsurface V_O_. Similarly, under multiple-V_O_ conditions in this study, we observed comparable migration behavior, extending beyond the two topmost layers. The unique migration behavior of subsurface V_O_’s to the third layer is attributed to the strong interaction between subsurface Ce^3+^ ions and third-layer V_O_’s, which facilitates additional lattice relaxation. This phenomenon aligns with interpretations presented in our recent work.^22^

Based on the simulations, the rate of polaron hopping dramatically increases with temperature from 300 to 900 K, whereas the rate of V_O_ migration reaches a maximum at 700 K and decreases at higher temperatures. To interpret this phenomenon, we introduce a simple model to qualitatively explain the entangled V_O_ migration and polaron hopping and utilize this model to estimate the temperature-dependent behavior of the V_O_ migration rate. Following Arrhenius’s theory, the probability of V_O_ migration to a specific site can be expressed as
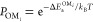
where Δ*E*_a_^OM_*i*_^ represents the activation energy barrier (in eV) for the *i*th V_O_ migration pathway. In a similar manner, the probability that a polaron hops from one site to another can be expressed as
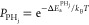
where Δ*E*_a_^PH_*j*_^ represents the activation energy barrier (in eV) for the *j*th polaron hopping pathway. According to the AIMD simulation, V_O_ migration is significantly slower than polaron hopping, implying that polarons may have hopped *N* times in the time it takes for a V_O_ migration to occur. During an energetically favorable V_O_ migration process, the polarons should not move; otherwise, this V_O_ migration pathway becomes energetically unfavorable. As illustrated in Figure 3, considering that there are *M* sites to which the polaron can hop and that the probability of choosing a hop pathway is *P*(*j*), the probability of the polaron not hopping is given by , and the probability that the polaron always maintains its position during one V_O_ migration process is . Therefore, the probability of the V_O_ migration considering the coupling of polarons hopping can be expressed as
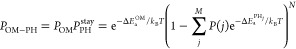


**Figure 3:**
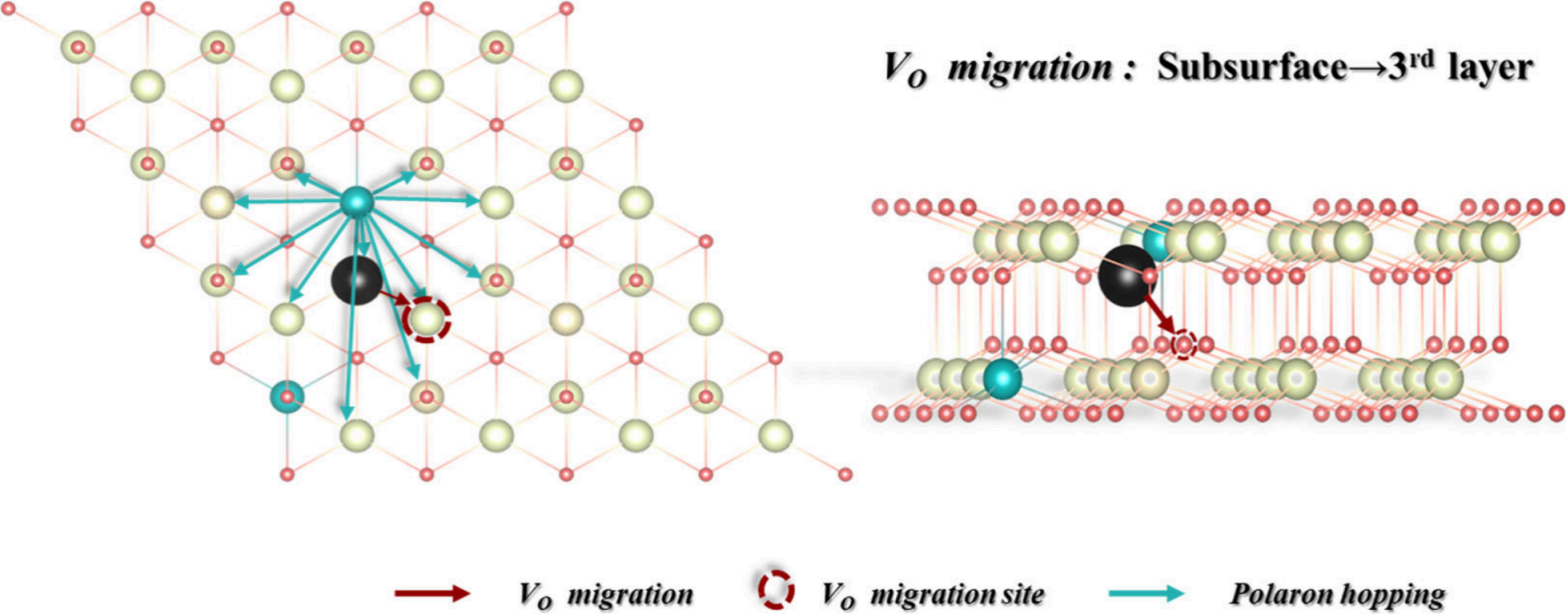
Entanglement between vacancy migration and polaron hopping. The V_O_ migrates from the subsurface to the third layer, as indicated by a brown arrow and circle, promoted by the presence of a NN Ce^3+^, while the polarons hop among sites surrounding the V_O_ (indicated by blue arrows). Ce^4+^, Ce^3+^, oxygen atoms, and the oxygen vacancy are colored white, blue, pink, and black, respectively.

We use the migration of a V_O_ from the subsurface to the third layer as an example to evaluate the V_O_ migration rates, as shown in Table 1. For polaron hopping, we assume that it prefers to take place among the sites around the V_O_, which consists of four sites in the NN cation shell and nine sites in the NNN cation shell. In total, there are 13 sites available for the polaron (Figure 3). However, when the V_O_ is formed, two sites are already occupied by polarons, leaving 11 sites vacant. Thus, we set *M* as 11 and all *P*(*j*) values equal to 1/11. At 700 K, the average migration time for the V_O_ is 359 fs while the average hopping time for polarons is 27 fs. As the temperature increases to 900 K, the migration time for the V_O_ increases to 368 fs while the hopping time for polarons is 28 fs. Here we assume the polaron makes approximately 13 hops for a V_O_ migration process, setting *N* to 13. For the sake of simplicity, we set the average value of Δ*E*_a_^OM^ ≈ 0.2 eV and Δ*E*_a_^PH_*j*_^ ≈ 0.15 eV according to DFT calculations (Figure 2 and Figure S6). Table 1 shows that, according to the classical Arrhenius theory, the probability of V_O_ migration and polaron hopping increases with temperature. However, when considering the entanglement between V_O_ migration and polaron hopping, the probability of vacancy migration does not increase monotonically with temperature. The highest probability for V_O_ migration occurs at 700 K and decreases as the temperature increases. This unusual V_O_ migration behavior is attributed to the rapid polaron hopping at high temperatures, which results in a residence time in positions favorable for facilitating migration being too short, thereby preventing V_O_ migration.

**Table 1:** Temperature-Dependent Probabilities of V_O_ Migration and Polaron Hopping

	300 K	500 K	700 K	900 K
*P*_OM_	0.00044	0.00968	0.03642	0.07604
*P*_OH_	0.00302	0.03077	0.08319	0.14457
*P*_OM–PH_	0.00042	0.00645	0.01177	0.00999

It is important to emphasize that while AIMD simulations provide critical phenomenological insights, systematic validation is necessary to determine whether the observed vacancy migration reflects genuine physical behavior rather than an artifact of the inherently limited time scales of AIMD approaches. To address this, we performed 10 ns molecular dynamics simulations using a neural network potential trained on the AIMD data (Figure 4A). The equilibration of these simulations was rigorously verified through potential energy convergence analysis and extended trajectory validation (Figures S7–S9). Four 10 ns trajectories were collected at 300, 500, 700, and 900 K, with typical structures analyzed statistically every 1 ps. As shown in Figure 4B, there were 3 (300 K), 250 (500 K), 487 (700 K), and 303 (900 K) V_O_ migrations. The frequency of V_O_ migration increases with temperature from 300 to 700 K and then decreases with a further temperature increase. These simulation outcomes corroborate the AIMD findings and the kinetic model, confirming the non-Arrhenius behavior persists even in the presence of multiple vacancies. To gain further insight into the distribution of V_O_’s, we plotted the number of vacancies in different oxygen layers, as shown in Figure 4C–F. At 300 K, V_O_’s predominantly migrate between the subsurface and the third layer, occasionally passing through the surface. However, with an increase in temperature, the presence of V_O_’s at the surface becomes infrequent, and they tend to occasionally migrate into the deeper layers.

**Figure 4:**
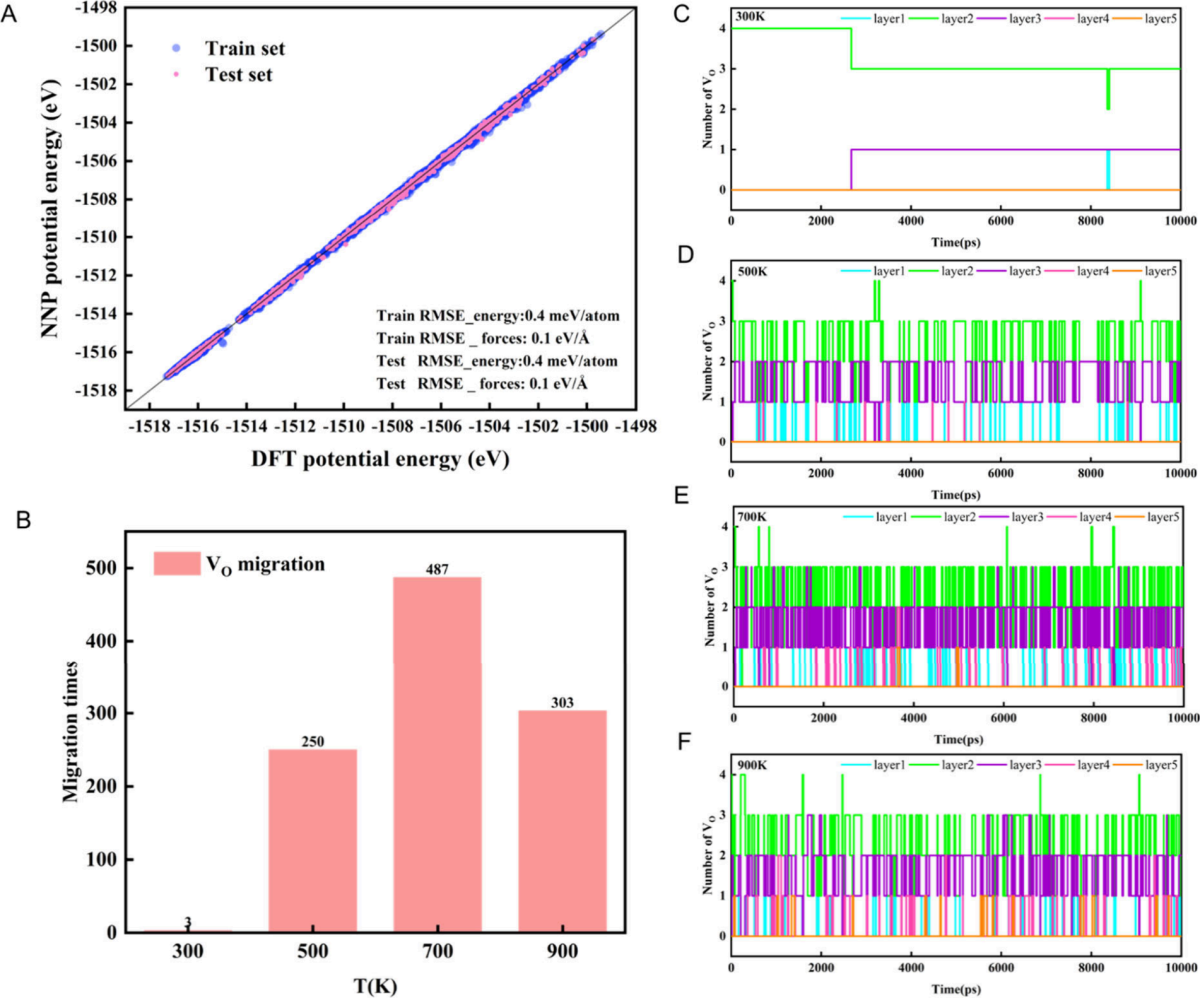
(A) Neural network potential (NNP) trained based on the AIMD trajectories. (B) Migration times of V_O_’s at different temperatures. (C–F) Trajectories of V_O_ migration at different temperatures from molecular dynamics simulations based on neural network force-field potential (statistics collected every 1 ps).

Our findings reveal a strong coupling between oxygen vacancy migration and polaron dynamics at the CeO_2_(111) surface. Despite the increased availability of migration pathways caused by higher vacancy concentrations, the process deviates from typical Arrhenius behavior, as vacancy migration probability does not increase monotonically with temperature. At increased temperatures, rapid polaron hopping disrupts vacancy migration, effectively hindering their mobility. Furthermore, vacancy migration is not confined to the surface and subsurface layers but frequently extends to the third oxygen layer, underscoring the complexity of vacancy transport in ceria. These insights deepen our understanding of the dynamic interplay between oxygen vacancies and polarons, offering valuable guidance for the design of metal oxide-based materials in applications spanning catalysis, energy conversion, and solid-state devices.

## Method

The spin-polarized DFT+*U* calculations with the Perdew–Burke–Ernzerhof (PBE) functional and AIMD simulations were conducted using the Vienna ab initio simulation package (VASP).^36−38^ To localize excess charges, a *U* value of 5.0 eV was applied to the Ce 4f states.^39−41^ Valence states were considered for the Ce (5s, 5p, 6s, 4f, 5d) and O (2s, 2p) electrons, and projector-augmented wave (PAW) potentials were employed to represent core–valence interactions.^42,43^ A plane wave cutoff of 400 eV was used to expand the Kohn–Sham valence states. The CeO_2_(111) surface was modeled as a periodic slab with a 4 × 4 surface supercell and included four O–Ce–O trilayers. To avoid interactions between periodic images, a vacuum layer of 15 Å was included. The bottom three atomic layers (O–Ce–O) were held fixed to their bulk positions, and the Γ point was adopted for all calculations. The reduced CeO_2_(111) surface was initially modeled with four oxygen vacancies in the subsurface layer. AIMD simulations were performed with the canonical (*NVT*) ensemble at temperatures of 300, 500, 700, and 900 K. Four trajectories with a time step of 1 fs were run for 50 ps. The transition-state structures for V_O_ migration and polaron hopping were identified using the climbing image nudged-elastic band (CI-NEB) algorithm.^44^

The neural network potential was trained on a data set comprising 8000 configurations randomly selected from AIMD trajectories using the n2p2 package,^45^ which inherently includes polaron effects. Consequently, the influence of the polaron is naturally incorporated into the molecular dynamics (MD) simulation process. The data set contained atomic coordinates and their corresponding potential energies, with 90% of the data set used for training and the remaining 10% reserved for testing to validate the accuracy of the neural network. Using n2p2, the total potential energy was defined as the sum of atomic potential energies, which were trained via an atomic neural network using symmetry functions to represent the structural and chemical environment of each atom. The neural network architecture consisted of an input layer, two hidden layers with 15 nodes each, and an output layer. After 10 iterations, the root-mean-square error (RMSE) of energies converged to 0.4 meV/atom and the RMSE of forces converged to 0.1 eV/Å. The trained neural network potential (NNP) was then employed for MD simulations using the Large-scale Atomic/Molecular Massively Parallel Simulator (LAMMPS).^46^ The simulations extended to 10 ns with time steps of 1 fs, employing the *NVT* ensemble at different temperatures (300, 500, 700, and 900 K).

## Supporting Information Available

Polaron hopping, positional relationship between the V_O_ and Ce^3+^, activation energy barrier of V_O_ migration and polaron hopping, details of V_O_ migration from the subsurface to the third layer, plot of the potential energy versus time, and oxygen vacancy migration behavior from the MD simulation based on a neural network (PDF)

## References

[ref1] Eguchi K.; Setoguchi T.; Inoue T.; Arai H. Electrical-Properties of Ceria-based Oxides and their Application to Solid Oxide Fuel-Cells. Solid State Ion. 1992, 52 (1–3), 165–172. 10.1016/0167-2738(92)90102-U.

[ref2] Park S.; Vohs J. M.; Gorte R. J. Direct Oxidation of Hydrocarbons in a Solid-Oxide Fuel Cell. Nature 2000, 404 (6775), 265–267. 10.1038/35005040. 10749204

[ref3] Miranda E.; Kano S.; Dou C.; Kakushima K.; Suñé J.; Iwai H. Nonlinear Conductance Quantization Effects in CeO_x_/SiO_2_-based Resistive Switching Devices. Appl. Phys. Lett. 2012, 101 (1), 012910 10.1063/1.4733356.

[ref4] Kašpar J.; Fornasiero P.; Graziani M. Use of CeO2-based Oxides in the Three-way Catalysis. Catal. Today 1999, 50 (2), 285–298. 10.1016/S0920-5861(98)00510-0.

[ref5] Monte R. D.; Kašpar J. On the Role of Oxygen Storage in Three-Way Catalysis. Topic. Catal. 2004, 28 (1), 47–57. 10.1023/B:TOCA.0000024333.08447.f7.

[ref6] Trovarelli A.; Boaro M.; Rocchini E.; de Leitenburg C.; Dolcetti G. Some Recent Developments in the Characterization of Ceria-based Catalysts. J. Alloy. Compd. 2001, 323–324, 584–591. 10.1016/S0925-8388(01)01181-1.

[ref7] Yashima M. Invited Review: Some Recent Developments in the Atomic-scale Characterization of Structural and Transport Properties of Ceria-based Catalysts and Ionic Conductors. Catal. Today 2015, 253, 3–19. 10.1016/j.cattod.2015.03.034.

[ref8] Deluga G. A.; Salge J. R.; Schmidt L. D.; Verykios X. E. Renewable Hydrogen from Ethanol by Autothermal Reforming. Science 2004, 303 (5660), 993–997. 10.1126/science.1093045. 14963325

[ref9] Xu C.; Qu X. Cerium Oxide Nanoparticle: A Remarkably Versatile Rare Earth Nanomaterial for Biological Applications. NPG Asia Mater. 2014, 6 (3), e90 10.1038/am.2013.88.

[ref10] Pulido-Reyes G.; Rodea-Palomares I.; Das S.; Sakthivel T. S.; Leganes F.; Rosal R.; Seal S.; Fernández-Piñas F. Untangling the Biological Effects of Cerium Oxide Nanoparticles: the Role of Surface Valence States. Sci. Rep. 2015, 5 (1), 15613 10.1038/srep15613. 26489858 PMC4615008

[ref11] Esch F.; Fabris S.; Zhou L.; Montini T.; Africh C.; Fornasiero P.; Comelli G.; Rosei R. Electron Localization Determines Defect Formation on Ceria Substrates. Science 2005, 309 (5735), 752–755. 10.1126/science.1111568. 16051791

[ref12] Torbrügge S.; Reichling M.; Ishiyama A.; Morita S.; Custance Ó. Evidence of Subsurface Oxygen Vacancy Ordering on Reduced CeO_2_(111). Phys. Rev. Lett. 2007, 99 (5), 056101 10.1103/PhysRevLett.99.056101. 17930771

[ref13] Ganduglia-Pirovano M. V.; Da Silva J. L. F.; Sauer J. Density-Functional Calculations of the Structure of Near-Surface Oxygen Vacancies and Electron Localization on CeO_2_(111). Phys. Rev. Lett. 2009, 102 (2), 026101 10.1103/PhysRevLett.102.026101. 19257295

[ref14] Li H.-Y.; Wang H.-F.; Gong X.-Q.; Guo Y.-L.; Guo Y.; Lu G.; Hu P. Multiple Configurations of the Two Excess 4*f* Electrons on Defective CeO_2_(111): Origin and Implications. Phys. Rev. B 2009, 79 (19), 193401 10.1103/PhysRevB.79.193401.

[ref15] Jerratsch J.-F.; Shao X.; Nilius N.; Freund H.-J.; Popa C.; Ganduglia-Pirovano M. V.; Burow A. M.; Sauer J. Electron Localization in Defective Ceria Films: A Study with Scanning-Tunneling Microscopy and Density-Functional Theory. Phys. Rev. Lett. 2011, 106 (24), 246801 10.1103/PhysRevLett.106.246801. 21770589

[ref16] Murgida G. E.; Ganduglia-Pirovano M. V. Evidence for Subsurface Ordering of Oxygen Vacancies on the Reduced CeO_2_(111) Surface Using Density-Functional and Statistical Calculations. Phys. Rev. Lett. 2013, 110 (24), 246101 10.1103/PhysRevLett.110.246101. 25165940

[ref17] Kullgren J.; Wolf M. J.; Castleton C. W. M.; Mitev P.; Briels W. J.; Hermansson K. Oxygen Vacancies versus Fluorine at CeO_2_(111): A Case of Mistaken Identity?. Phys. Rev. Lett. 2014, 112 (15), 156102 10.1103/PhysRevLett.112.156102. 24785057

[ref18] Wu X.-P.; Gong X.-Q. Clustering of Oxygen Vacancies at CeO_2_(111): Critical Role of Hydroxyls. Phys. Rev. Lett. 2016, 116 (8), 086102 10.1103/PhysRevLett.116.086102. 26967428

[ref19] Han Z.-K.; Yang Y.-Z.; Zhu B.; Ganduglia-Pirovano M. V.; Gao Y. Unraveling the Oxygen Vacancy Structures at the Reduced CeO_2_(111) Surface. Phys. Rev. Mater. 2018, 2, 035802 10.1103/PhysRevMaterials.2.035802.

[ref20] Han Z.-K.; Zhang L.; Liu M.; Ganduglia-Pirovano M. V.; Gao Y. The Structure of Oxygen Vacancies in the Near-Surface of Reduced CeO_2_(111) Under Strain. Front. Chem. 2019, 7, 436 10.3389/fchem.2019.00436. 31275923 PMC6592146

[ref21] Pérez-Bailac P.; Lustemberg P. G.; Ganduglia-Pirovano M. V. Facet-dependent Stability of Near-Surface Oxygen Vacancies and Excess Charge Localization at CeO_2_ Surfaces. J. Phys.: Condens. Matter. 2021, 33, 504003 10.1088/1361-648X/ac238b. 34479232

[ref22] Zhang Y. J.; Han Z.-K.; Zhu B.; Hu X. J.; Troppenz M.; Rigamonti S.; Li H.; Draxl C.; Ganduglia-Pirovano M. V.; Gao Y. Decoupling Many-Body Interactions in CeO_2_ (111) Oxygen Vacancy Structure with Statistical-Learning and Cluster Expansion. Nanoscale 2025, 17, 4531–4542. 10.1039/D4NR04591B. 39801491

[ref23] Sutton J. E.; Beste A.; Overbury S. H. Origins and Implications of the Ordering of Oxygen Vacancies and Localized Electrons on Partially Reduced CeO_2_(111). Phys. Rev. B 2015, 92 (14), 144105 10.1103/PhysRevB.92.144105.

[ref24] Zhang L.; Sun L.; Meng Q.; Wu J.; Hao X.; Zhai S.; Dou W.; Jia Y.; Zhou M. Strain-Engineered Formation, Migration, and Electronic Properties of Polaronic Defects in CeO_2_. Phys. Status Solidi B 2021, 258 (6), 2100020 10.1002/pssb.202100020.

[ref25] Zacherle T.; Schriever A.; De Souza R. A.; Martin M. Ab Initio Analysis of the Defect Structure of Ceria. Phys. Rev. B 2013, 87 (13), 134104 10.1103/PhysRevB.87.134104.

[ref26] Sun L.; Huang X.; Wang L.; Janotti A. Disentangling the Role of Small Polarons and Oxygen Vacancies in CeO_2_(111). Phys. Rev. B 2017, 95 (24), 245101 10.1103/PhysRevB.95.245101.

[ref27] Richter N. A.; Sicolo S.; Levchenko S. V.; Sauer J.; Scheffler M. Concentration of Vacancies at Metal-Oxide Surfaces: Case Study of MgO(100). Phys. Rev. Lett. 2013, 111 (4), 045502 10.1103/PhysRevLett.111.045502. 23931382

[ref28] Tuller H. L.; Nowick A. S. Small Polaron Electron Transport in Reduced CeO_2_ Single Crystals. J. Phys. Chem. Solids 1977, 38 (8), 859–867. 10.1016/0022-3697(77)90124-X.

[ref29] Namai Y.; Fukui K.-i.; Iwasawa Y. Atom-Resolved Noncontact Atomic Force Microscopic Observations of CeO_2_(111) Surfaces with Different Oxidation States: Surface Structure and Behavior of Surface Oxygen Atoms. J. Phys. Chem. B 2003, 107 (42), 11666–11673. 10.1021/jp030142q.

[ref30] Namai Y.; Fukui K.-I.; Iwasawa Y. Atom-Resolved Noncontact Atomic Force Microscopic and Scanning Tunneling Microscopic Observations of the Structure and Dynamic Behavior of CeO_2_(111) Surfaces. Catal. Today 2003, 85 (2), 79–91. 10.1016/S0920-5861(03)00377-8.

[ref31] Li H.-Y.; Wang H.-F.; Guo Y.-L.; Lu G.-Z.; Hu P. Exchange between Sub-Surface and Surface Oxygen Vacancies on CeO_2_(111): A New Surface Diffusion Mechanism. Chem. Commun. 2011, 47 (21), 6105–6107. 10.1039/c1cc11226k. 21523265

[ref32] Plata J. J.; Márquez A. M.; Sanz J. F. Transport Properties in the CeO_2-x_(111) Surface: From Charge Distribution to Ion-Electron Collaborative Migration. J. Phys. Chem. C 2013, 117 (48), 25497–25503. 10.1021/jp4066532.

[ref33] Su Y.-Q.; Filot I. A. W.; Liu J.-X.; Tranca I.; Hensen E. J. M. Charge Transport over the Defective CeO_2_(111) Surface. Chem. Mater. 2016, 28 (16), 5652–5658. 10.1021/acs.chemmater.6b01548.

[ref34] Zhang D.; Han Z.-K.; Murgida G. E.; Ganduglia-Pirovano M. V.; Gao Y. Oxygen-Vacancy Dynamics and Entanglement with Polaron Hopping at the Reduced CeO_2_(111) Surface. Phys. Rev. Lett. 2019, 122 (9), 096101 10.1103/PhysRevLett.122.096101. 30932558

[ref35] Paul M.; Karmakar S.; Satpati B.; Chakraborty S. Temperature dependent polaronic contribution on conduction mechanism in ceria-based devices. Phys. B 2023, 666, 415098 10.1016/j.physb.2023.415098.

[ref36] Kresse G.; Furthmüller J. Efficient Iterative Schemes for Ab Initio Total-Energy Calculations Using a Plane-Wave Basis Set. Phys. Rev. B 1996, 54 (16), 11169–11186. 10.1103/PhysRevB.54.11169. 9984901

[ref37] Perdew J. P.; Burke K.; Ernzerhof M. Generalized Gradient Approximation Made Simple. Phys. Rev. Lett. 1996, 77 (18), 3865–3868. 10.1103/PhysRevLett.77.3865. 10062328

[ref38] Kresse G.; Furthmüller J. Efficiency of Ab-Initio Total Energy Calculations for Metals and Semiconductors Using a Plane-Wave Basis Set. Comput. Mater. Sci. 1996, 6 (1), 15–50. 10.1016/0927-0256(96)00008-0.

[ref39] Dudarev S. L.; Botton G. A.; Savrasov S. Y.; Humphreys C. J.; Sutton A. P. Electron-Energy-Loss Spectra and the Structural Stability of Nickel Oxide: An LSDA+U Study. Phys. Rev. B 1998, 57 (3), 1505–1509. 10.1103/PhysRevB.57.1505.

[ref40] Nolan M.; Grigoleit S.; Sayle D. C.; Parker S. C.; Watson G. W. Density Functional Theory Studies of the Structure and Electronic Structure of Pure and Defective Low Index Surfaces of Ceria. Surf. Sci. 2005, 576 (1), 217–229. 10.1016/j.susc.2004.12.016.

[ref41] Castleton C. W. M.; Kullgren J.; Hermansson K. Tuning LDA+U for Electron Localization and Structure at Oxygen Vacancies in Ceria. J. Chem. Phys. 2007, 127 (24), 244704 10.1063/1.2800015. 18163692

[ref42] Blöchl P. E. Projector Augmented-Wave Method. Phys. Rev. B 1994, 50 (24), 17953–17979. 10.1103/PhysRevB.50.17953. 9976227

[ref43] Kresse G.; Joubert D. From Ultrasoft Pseudopotentials to the Projector Augmented-Wave Method. Phys. Rev. B 1999, 59 (3), 1758–1775. 10.1103/PhysRevB.59.1758.

[ref44] Henkelman G.; Uberuaga B. P.; Jónsson H. A Climbing Image Nudged Elastic Band Method for Finding Saddle Points and Minimum Energy Paths. J. Chem. Phys. 2000, 113 (22), 9901–9904. 10.1063/1.1329672.

[ref45] Singraber A.; Behler J.; Dellago C. Library-Based *LAMMPS* Implementation of High-Dimensional Neural Network Potentials. J. Chem. Theory Comput. 2019, 15, 1827–1840. 10.1021/acs.jctc.8b00770. 30677296

[ref46] Thompson A. P.; Aktulga H. M.; Berger R.; Bolintineanu D. S.; Brown W. M.; Crozier P. S.; in ’t Veld P. J.; Kohlmeyer A. S.; Moore G.; Nguyen T. D.; Shan R.; Stevens M. J.; Tranchida J.; Trott C.; Plimpton S. J. LAMMPS - a flexible simulation tool for particle-based materials modeling at the atomic, meso, and continuum scales. Comput. Phys. Commun. 2022, 271, 108171 10.1016/j.cpc.2021.108171.

