# Supplementary material for: Revealing the Non-Arrhenius Migration of Oxygen Vacancies at the CeO2(111) Surface
Source: J Phys Chem Lett. 2025 Apr 6;16(15):3734–40. doi: 10.1021/acs.jpclett.5c00444 (PMC12010425; doi:10.1021/acs.jpclett.5c00444)
Supplement: Supplementary file 1 — jz5c00444_si_001.pdf [file jz5c00444_si_001.pdf]

Supporting Information

Revealing the Non-Arrhenius Migration of Oxygen Vacancies at the CeO_2(111) Surface

Yujing Zhang[1,2], Huabing Cai[2,3], Beien Zhu[2,5], Zhong-Kang Han[3], Hui Li[1], M. Verónica Ganduglia-Pirovano[4]*, Yi Gao[2,5]**

[1]Beijing Advanced Innovation Center for Soft Matter Science and Engineering, Beijing University of Chemical Technology, Beijing 100029, China

[2]Photon Science Research Center for Carbon Dioxide, Shanghai Advanced Research Institute, Chinese Academy of Sciences, Shanghai 201210, China

[3]School of Materials Science and Engineering, Zhejiang University, Hangzhou 310027, China

[4]Instituto de Catálisis y Petroleoquímica, Consejo Superior de Investigaciones Científicas, 28049 Madrid, Spain

[5]Key Laboratory of Low-Carbon Conversion Science & Engineering, Shanghai Advanced Research Institute, Chinese Academy of Sciences, Shanghai 201210, China

Email: hli@buct.edu.cn; vgp@icp.csic.es; gaoyi@sari.ac.cn

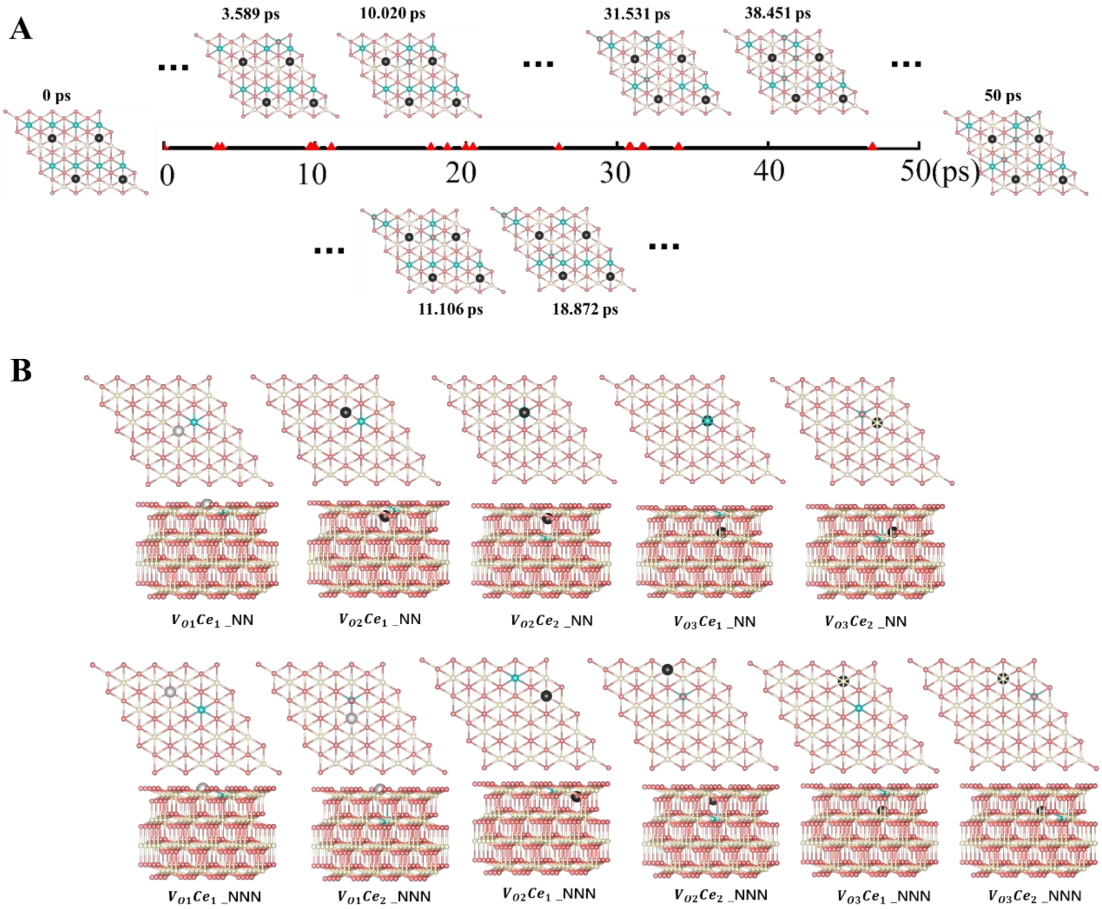

Figure S1. A. Polaron hopping at 300 K. B. Nearest Neighbor (NN) and Next-Nearest Neighbor (NNN) positional relationship between a V_O and a Ce^{3+}, denoted as $V_{On}Ce_m$_NN(NNN), where n and m denote the oxygen atomic layer and the Ce atomic layer where the V_O and Ce^{3+} are located, respectively. Ce^{4+}, Ce^{3+}, oxygen atoms, and surface oxygen vacancy are depicted in white, blue, pink, and light gray, respectively, while subsurface and third oxygen layer vacancies are shown in black. This color scheme is applied throughout all subsequent figures.

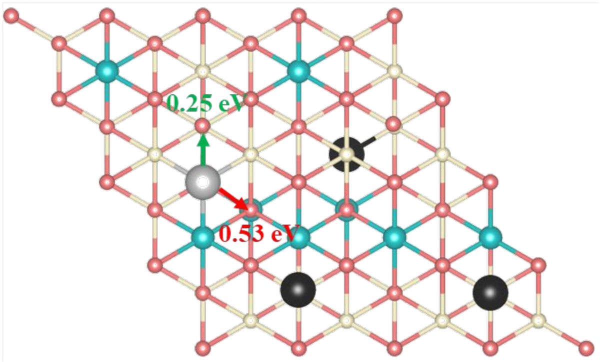

Ce^{4+}-Ce^{4+} : 0.25 eV
Ce^{3+}-Ce^{4+} : 0.53 eV

Figure S2. Migration of a V_O from the surface to the subsurface through a Ce^{4+}–Ce^{4+} bridge and a Ce^{3+}–Ce^{4+} bridge.

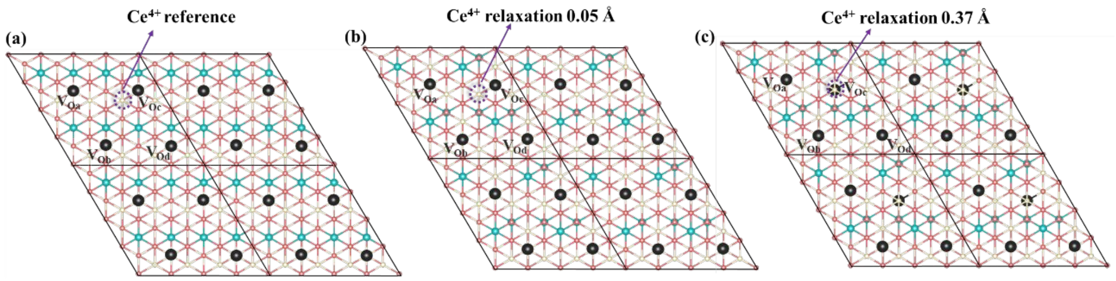

$$\textit{relaxation}\ \text{distance}=\sqrt{(x_j-x_i)^2+(y_j-y_i)^2+(z_j-z_i)^2}$$

Figure S3. (a) Top view of the pristine surface. (b) Initial state and (c) final state (c) for the migration of V_{Oc} from the subsurface layer to third oxygen layer. The purple dashed circle is Ce^{4+} ion, indicating the relaxation distances of the Ce^{4+} ion in the initial and final states with respect to the pristine surface.

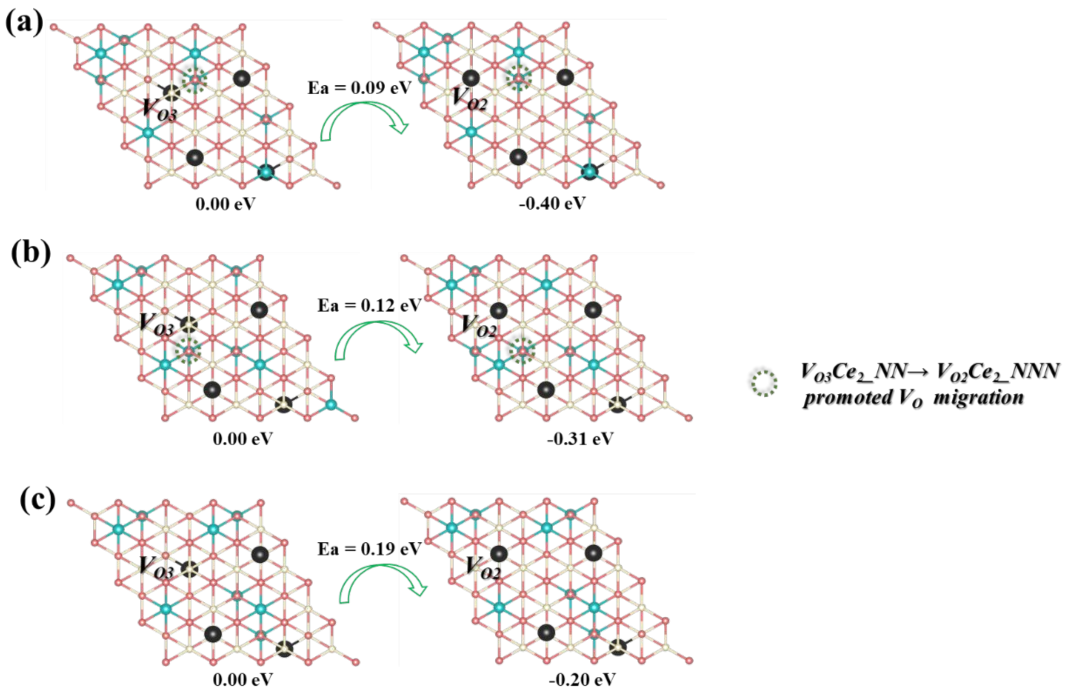

Figure S4. V_O migration pathways from the third oxygen layer (V_{O3}) to the subsurface (V_{O2}). Green dotted circles indicate the subsurface Ce^{3+} (Ce_2). The paths in (a) and (b) correspond to a $V_{O3}Ce_2$_NN → $V_{O2}Ce_2$_NNN migration via the NN-polaron-promoted mechanism. $V_{On}Ce_m$_NN(NNN) denotes the positional relationship between a V_O and a Ce^{3+}, indicating whether they are nearest (NN) or next-nearest neighbors (NNN). Here, n and m represent the oxygen atomic layer and the Ce atomic layer where the V_O and Ce^{3+} are located, respectively. In the initial state, a subsurface Ce^{3+} is in the second cationic plane (Ce_2) is a NN to a third oxygen layer V_O (V_{O3}). In the final state, the vacancy migrates to the subsurface layer (V_{O2}), and the Ce^{3+} shifts to a NNN

position.

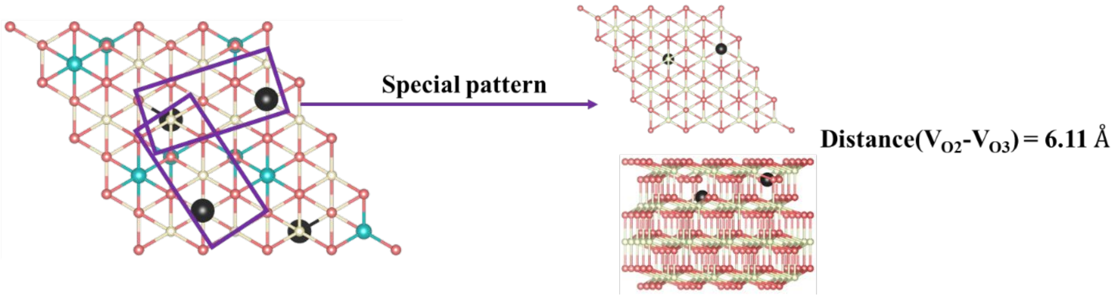

Figure S5. Unique pattern formed by a V_O in the third layer (V_{O3}) and a V_O in the subsurface layer (V_{O2}), separated by a distance of 6.11 Å. This distance corresponds to the fifth V_O–V_O neighbor distance in bulk CeO_2.

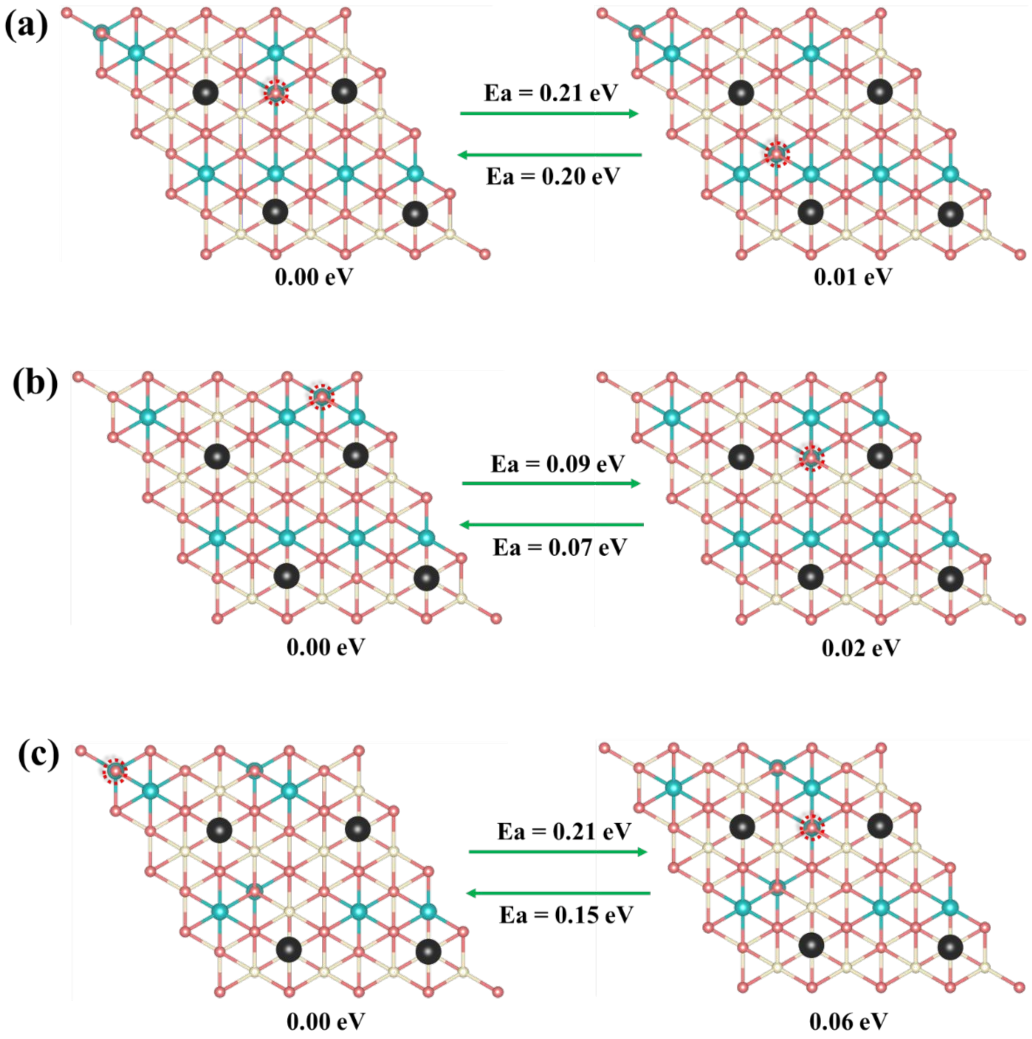

Figure S6. Activation energy barriers associated with polaron hopping.

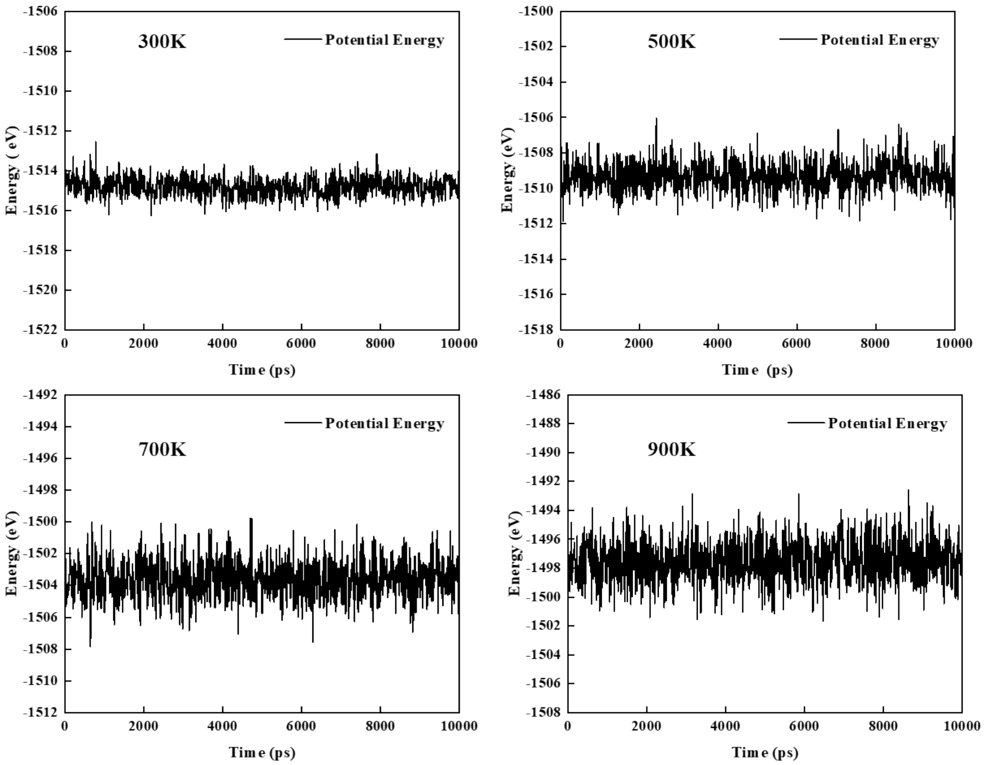

Figure S7. Potential energy versus time plot of the initial 10 ns NNP-MD simulation.

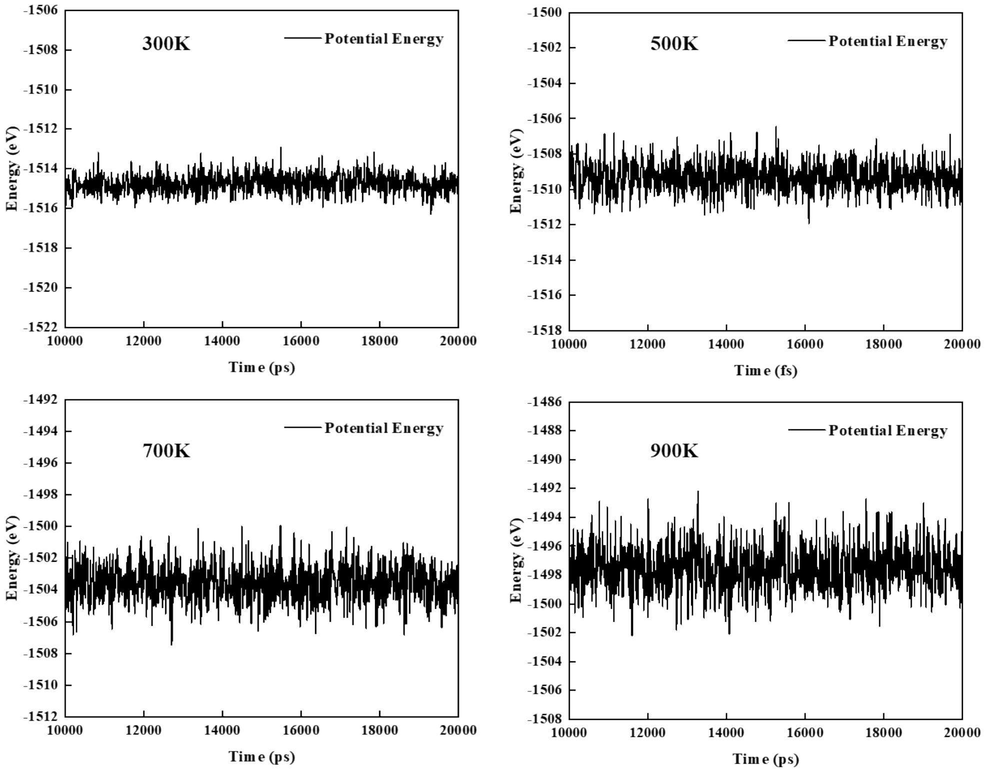

Figure S8. Potential energy versus time plot of the additional 10 ns NNP-MD simulation.

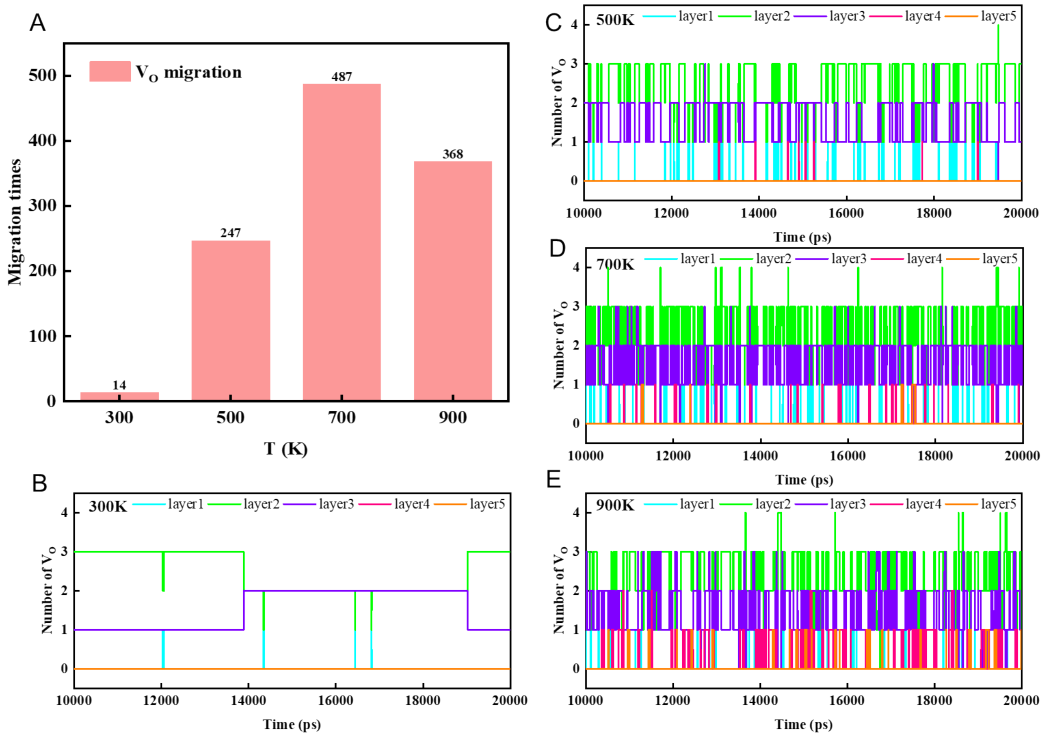

Figure S9. V_O's migration behavior of the additional 10 ns NNP-MD simulation. A: Migration times of V_O's at different temperatures. B-E: Trajectories of V_O migration at different temperatures from MD simulation using a neural network force-field potential, with statistical data collected every 1ps.
